# Cell Culture Process Scale-Up Challenges for Commercial-Scale Manufacturing of Allogeneic Pluripotent Stem Cell Products

**DOI:** 10.3390/bioengineering9030092

**Published:** 2022-02-25

**Authors:** Brian Lee, Sunghoon Jung, Yas Hashimura, Maximilian Lee, Breanna S. Borys, Tiffany Dang, Michael S. Kallos, Carlos A. V. Rodrigues, Teresa P. Silva, Joaquim M. S. Cabral

**Affiliations:** 1 PBS Biotech, Inc., Camarillo, CA 93012, USA; sjung@pbsbiotech.com (S.J.); yhashimura@pbsbiotech.com (Y.H.); mlee@pbsbiotech.com (M.L.); bborys@pbsbiotech.com (B.S.B.); 2 Department of Biomedical Engineering, Schulich School of Engineering, University of Calgary, 2500 University Dr. NW, Calgary, AB T2N 1N4, Canada; tiffany.dang@ucalgary.ca (T.D.); mskallos@ucalgary.ca (M.S.K.); 3 iBB–Institute for Bioengineering and Biosciences and Department of Bioengineering, Instituto Superior Técnico, Universidade de Lisboa, 1049-001 Lisboa, Portugal; carlos.rodrigues@tecnico.ulisboa.pt (C.A.V.R.); teresasilva@tecnico.ulisboa.pt (T.P.S.); joaquim.cabral@tecnico.ulisboa.pt (J.M.S.C.)

**Keywords:** allogeneic cell therapy, induced pluripotent stem cell, human embryonic stem cell, cell aggregate, expansion, differentiation, scalable manufacturing, scale up, single-use bioreactor, vertical-wheel, computational fluid dynamics, shear stress, turbulent energy dissipation rate, homogeneous hydrodynamic environment, oxygenation, medium exchange

## Abstract

Allogeneic cell therapy products, such as therapeutic cells derived from pluripotent stem cells (PSCs), have amazing potential to treat a wide variety of diseases and vast numbers of patients globally. However, there are various challenges related to manufacturing PSCs in single-use bioreactors, particularly at larger volumetric scales. This manuscript addresses these challenges and presents potential solutions to alleviate the anticipated bottlenecks for commercial-scale manufacturing of high-quality therapeutic cells derived from PSCs.

## 1. Introduction

With their potential to cure a wide variety of disease indications and address vast patient populations, allogeneic cell therapies derived from pluripotent stem cells (PSCs) are poised to revolutionize therapeutic medicines [1,2]. However, 2D planar manufacturing technologies that have been commonly used for small-scale R&D and early-stage clinical trials are inadequate and cost-prohibitive for production at the larger scales required for late-stage clinical trials and commercial manufacturing [3]. Single-use bioreactors are widely recognized as a feasible manufacturing solution to meet the unique process requirements of PSCs [4]. One of the challenges for future success in commercializing allogeneic cell therapy products is establishing a scalable manufacturing technology that can reliably reproduce the yield and quality of PSC-derived products generated from small-scale R&D methods at larger scales sufficient for commercial manufacturing [5].

PSCs are mortal human cells that include specific cell types such as human embryonic stem cells (hESCs) and induced pluripotent stem cells (iPSCs). When cultivated in a 2D planar vessel, PSCs attach to a surface substrate and grow as a monolayer. In contrast, starting from single cells or small clumps in suspension culture, PSCs will naturally form spherical cell aggregates either through clumping or cell division in the absence of culture substrates such as microcarriers [6]. The formation of cell aggregates is required for the cell expansion phase and subsequent differentiation, which can be a multi-step process that directs the pluripotent cells to turn into a final target cell type for treating a particular disease.

Various types of vessels have been used for aggregate formation for small-scale research or process development purposes. Ultra-low attachment well-plates and dishes can be seeded with single cells or cell clumps and then rotated on an orbital shaker inside an incubator. However, this method can result in cells experiencing inconsistent gas transfer rates and hydrodynamic forces, resulting in unwanted aggregate heterogeneity. AggreWell^TM^ plates (STEMCELL Technologies, Vancouver, BC, USA), which use centrifugal force to distribute single cells into microwells shaped like inverted pyramids, could be used as an alternative option to promote the formation of more homogeneous aggregates. However, using any kind of well-plate for large-scale manufacturing, either by scale up or scale out, can quickly become cost-prohibitive in regard to consumables, labor, and time. Shaker flasks or roller bottles provide a suspension-based mixing environment. However, their lack of process controls and scalability compared to bioreactors can also result in heterogeneous aggregates and make them cost-prohibitive for large-scale manufacturing.

A robust and scalable manufacturing process is crucial for the consistent production of PSC aggregates that will become allogeneic cell therapy products for patients. For a typical process, initial donor cells, often of limited quantity, first go through a seed train expansion process before being transferred into larger-scale platforms such as bioreactors for various cell culture processes. Progressively larger bioreactors can be used sequentially to generate the vast cell quantities necessary for allogeneic applications. A single dose of therapeutic cell therapy for one patient could require billions of cells [7]. For example, if approximately one million cells per milliliter of medium is the target concentration for PSCs in a bioreactor, then one dose will typically require one liter of medium at the time of bioreactor harvest. Therefore, for an allogeneic cell manufacturing process, hundred or thousand liters of culture working volume should be required to generate hundred or thousand of doses per batch. Furthermore, a certain percentage of cells will invariably be lost during various downstream processes such as harvest, wash, concentration, formulation, and fill/finish steps. The target number of cells produced per lot will need to account for these expected losses.

This communication will highlight specific manufacturing processes that may be particularly challenging, especially at larger volumetric scales, and provide examples of how optimal technology can provide solutions to enable scalable production of allogeneic cell therapies.

## 2. Generating High Quality Seed Culture

While vast quantities of therapeutic cells will need to be produced for allogeneic applications, the proprietary, original donor cells are usually limited in supply and frozen in vials for storage. A reliable seed train process to generate high-quality seed cells from finite donor cells is an important first step for scalable manufacturing in bioreactors. 

Typically, 2D planar vessels such as multi-well plates or T-flasks are used to expand cells from a working cell bank (WCB) vial of donor cells. The cell expansion process may scale up and continue in a larger planar vessel such as a T-flask, roller bottles, or a multilayer plate, e.g., Cell Factory^®^ (Thermo Scientific, Waltham, MA, USA) or CellSTACK^®^ (Corning, Corning, NY, USA), before cells enter a terminal bioreactor (at the production stage). However, the limited scalability of 2D platforms restricts manufacturing to a scale-out approach (as opposed to scale-up) to generate enough cells to inoculate larger volume bioreactors. The limitations of 2D platforms for generating a large number of cells, based on various aspects such as labor and material costs as well as difficulties with operational efficiency, have been well-documented [8]. Specifically, unit operations involving numerous 2D planar vessels require prolonged handling and processing times with many operators, which can negatively impact cell quality, process reliability, and cost-effectiveness. 

A cell culture procedure that works at a small scale often requires engineering-based development work for successful scale-up to larger volumes. The scale-up development process can be greatly facilitated through the use of the same or similar manufacturing platforms instead of having to create different procedures due to different mixing technologies at different scales (e.g., 2D wells at small scale and 3D bioreactors at large scale, or wave-motion bioreactors at small scale and stirred-impeller mixing at large scale).

Ideally, a single type of scalable manufacturing platform such as a suspension bioreactor could be used for the entire manufacturing process, starting even from a WCB vial. Alternatively, a smaller, scale-down model bioreactor could be used as an intermediate seed train (i.e., between an initial 2D planar vessel-based seed train and the terminal bioreactor). It is crucial to consistently generate high-quality seed cells, regardless of the use of 2D planar vessels, small-scale bioreactors, or a combination of both, before proceeding to large-scale manufacturing. 

## 3. Scale up of Optimal Hydrodynamic Conditions

While single-use bioreactors can become the standard manufacturing platform for allogeneic cell therapies, various types of therapeutic cells may be sensitive to the bioreactor mixing characteristics in different ways. In particular, the hydrodynamic conditions within a bioreactor will significantly impact the biological performance of PSCs and the resulting efficiency of cell culture processes such as expansion and differentiation [9,10]. Anchorage-dependent cells grown on microcarriers or as aggregates are more sensitive to hydrodynamic shear stress than those grown as single cells in suspension bioreactors. Examples of cell types that can be cultured as aggregates include PSCs, neural stem cells [11], and mammary epithelial stem cells [12]. In contrast, hematopoietic stem/progenitor cells and immune cells like T cells and NK cells are typically grown as single cells or loose clumps [13].

The morphology of cell aggregates in a bioreactor can greatly influence the efficiency and production yield of expansion and differentiation processes [14,15,16]. A homogeneous distribution of spherical aggregates, with the optimal diameter dependent on cell type, is ideal for consistent and uniform distribution of nutrients, growth factors, and gases throughout the entirety of every aggregate. If an aggregate is misshapen or even too large of a sphere, nutrients and gasses may be unable to evenly diffuse from the aggregate’s surface to its center, leading to necrosis in the center or heterogeneous cell populations during expansion or differentiation [17,18]. Aggregates that are still spherical but too small in diameter due to hydrodynamic conditions may not have diffusion issues but can produce suboptimal yields for expansion and differentiation processes [19]. 

While PSC aggregate formation can be influenced by variables such as cell proliferation rate, cell–cell adhesion strength, and cell packing density, the hydrodynamic environment inside a bioreactor, which is created by the impeller used to continually mix the liquid medium, also has a significant impact on determining the aggregate size and, ultimately, cell viability [20]. The hydrodynamic environment can be characterized by parameters such as fluid flow pattern, the distribution of turbulent energy dissipation rate (EDR), and the magnitude of fluid shear force [21]. 

After the initial seeding of single cells or small clumps into a bioreactor, collisions due to the hydrodynamic environment will facilitate aggregate growth, either through the addition of additional single cells or small clumps onto existing aggregates or the fusion of small aggregates into larger ones [22]. At sufficiently higher agitation rates, the increased levels of shear stress will promote the breakage of loosely attached or temporarily agglomerated larger aggregates and thus limit their maximum possible size [10]. 

**Figure 1:**
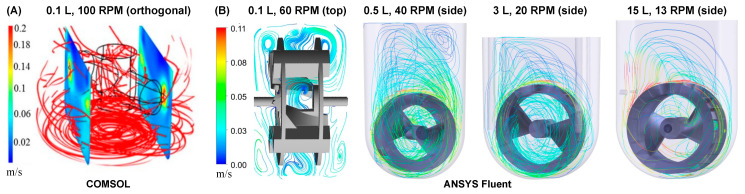
CFD models of velocity streamlines for (**A**) horizontal-blade spinner and (**B**) VW bioreactors. All agitation rates are sufficient to fully suspend large particles such as cell aggregates. An identical modeling method was used for both fluid simulation softwares (COMSOL and ANSYS Fluent). Adapted from Borys et al. (2018) [27].

Fluid mixing in a bioreactor using a traditional horizontal-blade impeller creates a heterogeneous hydrodynamic environment [23]. The highest levels of turbulent EDR and shear forces will be near the tips of the rapidly spinning impeller, with decreasing gradients of these hydrodynamic parameters as the distance from the impeller increases [24,25]. Varying ranges of energy dissipation rates and fluid shear forces in a horizontal-blade impeller bioreactor result in a non-uniform hydrodynamic environment, which in turn causes a heterogeneous distribution of aggregate morphologies. In addition, fluid flows primarily in an axial direction with horizontal-blade impellers. Computational Fluid Dynamics (CFD) modeling of a horizontal-blade spinner shows predominantly radial, and high velocity streamlines underneath the impeller (black cylindrical outline) near the bottom of the vessel (Figure 1A). Furthermore, the perpendicular cut planes of velocity indicate regions of low (blue) velocity directly above and below the horizontal plane of the spinning impeller, with high velocities again near the impeller’s tips. These stratified sections of significantly different velocities can cause cells or cell aggregates to potentially become “trapped” in either high velocity “tornados” (around the impeller center plane) or low velocity “dead zones” (top and bottom of the vessel) instead of continuously circulating through all regions of the vessel [21,23]. These heterogeneous mixing conditions and resulting wide distribution of aggregate morphologies are exacerbated at larger scales for horizontal-blade bioreactors. 

In contrast, the fluid in a vertical-wheel (VW) bioreactor moves in a lemniscate pattern throughout the entire volume of the U-shaped vessel. CFD modeling shows that the unique VW impeller geometry of peripheral paddles and oppositely-oriented axial vanes combine radial mixing in the vertical plane and axial mixing in the horizontal plane (Figure 1B) even across various scales and agitation rates [26,27].

As the VW impeller rotates and generates the lemniscate fluid flow pattern, PSC aggregates will continually travel throughout the entirety of the vessel without becoming trapped or experiencing extreme changes in velocities or shear, even during scale-up. For a given volumetric scale of VW bioreactor, localized turbulence around the impeller’s circumference increases with higher agitation rates, but to a much lesser extent than the tips of a horizontal impeller.

**Figure 2:**
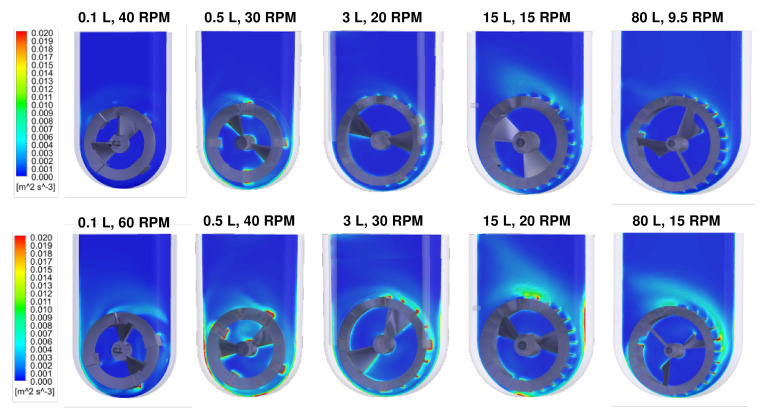
CFD models of EDR contours for various scales and agitation rates in VW bioreactors. A narrow distribution of EDR, which promotes formation of uniformly spherical aggregates, can be maintained during scale up in VW bioreactors. Adapted from Dang et al. (2021) [28].

CFD models also indicated that the distributions of turbulent EDR values in VW bioreactors are consistently narrow, even for different combinations of volume and agitation rates, with some representative examples shown in Figure 2 [28].

At a given scale, increasing the agitation rate results in more turbulent EDR around the impeller’s circumference, which can broaden the distribution of EDR but not by a significant order of magnitude. Most importantly, a narrow distribution of EDR (indicated by majority blue coloration) can be achieved at any scale of the VW bioreactor by optimizing the agitation rate. The velocity streamlines and narrow distributions of turbulent EDR confirm that VW bioreactors can provide a homogeneous mixing environment for PSC aggregates. Furthermore, optimal hydrodynamic conditions achieved at a small scale can be consistently maintained during scale up in VW bioreactors, which is required to develop the entire manufacturing process efficiently.

**Figure 3:**
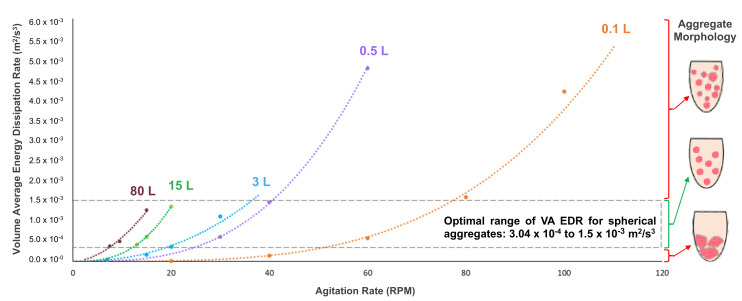
Curves to determine average EDR based on VW bioreactor working volume and agitation rate. Average EDR can be used to predict aggregate morphology for a particular combination of volume and agitation, with desired spherical aggregates as consequence of average EDR within an optimal range. Adapted from Dang et al. (2021) [28].

As stated previously, the mixing environment of a bioreactor will have a major effect on the biological performance of PSC aggregates. Spherical aggregates of an optimal range of diameter (depending on PSC type) are essential to maximizing cell yield and quality for various manufacturing processes. In order to confirm the relationship between the hydrodynamic condition of EDR and aggregate morphology, the average EDR was calculated (from CFD contour models) for different combinations of VW bioreactor volume and impeller agitation rate. When a VW bioreactor mixes a predetermined working volume of a medium at a steady agitation rate, EDR values are generated (typically within a narrow distribution). The mean of all those values is the average EDR for that particular combination of volume and agitation. Various data points of average EDR versus agitation rate were plotted and used to generate best-fit curves for each volume of the bioreactor (Figure 3) [28]. 

**Figure 4:**
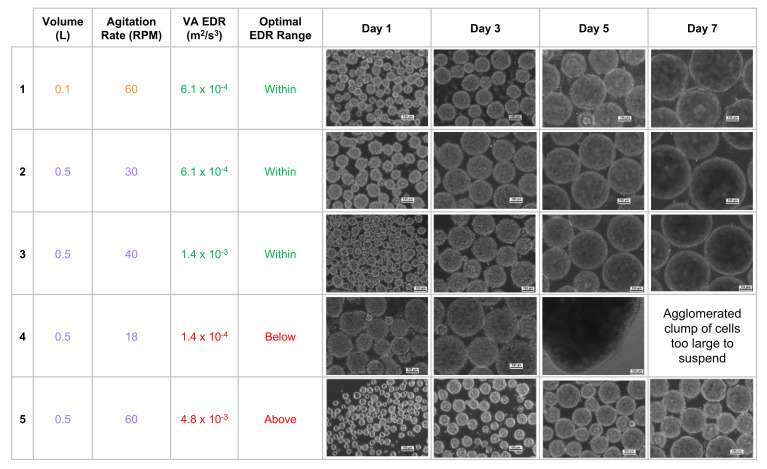
Observed morphologies of iPSC aggregates for different combinations of VW working volume and agitation rate. Uniformly spherical aggregates correspond to average EDRs that fall within the optimal range. There is also an inverse correlation between average EDR and aggregate size. Photomicrographs were taken at 10× magnification. Scale bars: 100 μm. Adapted from Dang et al. (2021) [28].

Furthermore, it was found that average EDR values that fell within an optimal range correlated to the formation of uniformly spherical PSC aggregates under those particular combinations of volume and agitation. The suggested average EDR range of 3.0 × 10^−4^ to 1.5 × 10^−3^ m^2^/s^3^ is based on results from multiple experiments and PSC lines [28]. Biological experiments confirmed that targeting an average EDR within the optimal range consistently produces a homogeneous distribution of spherical iPSC aggregates, even for different volume and agitation rate combinations. In contrast, average EDR values that were above or below the optimal range would result in heterogeneous aggregates that were too small or large, respectively (Figure 4).

The homogeneous distribution of spherical aggregates results from choosing a volume and agitation rate that results in an average EDR within the optimal range (Figure 4, rows 1–3). It was also observed that targeting an identical average EDR at different combinations of volume and agitation rates resulted in spherical aggregates of nearly identical diameter (Figure 4, rows 1 & 2). This phenomenon of nearly identical hydrodynamic conditions at different volumes is the key to the scalability of VW bioreactors. Biological experiments and process optimization can first be performed cost-effectively at a small scale to determine the parameters that produce an average EDR within the optimal zone and desired aggregate formation for a particular type of PSC. Once that target average EDR is known, the curves from Figure 3 can be used as a predictive tool to find the agitation rates at higher volumes to achieve the same target average EDR and promote spherical aggregate formation at larger scales. 

If the agitation rate is too low for a chosen volume (Figure 4, row 4), not only will the average EDR be below the optimal range, but the distribution of all EDR values will broaden, which in turn leads to a heterogeneous distribution of aggregates. In addition, lower agitation means weaker shear forces acting on the surface of aggregates, which likely allows for larger clumps to form. If sufficient clumping occurs, then aggregate may become too heavy to remain suspended in the medium at that agitation rate. Having an agitation rate that is too high leads to an average EDR above the optimal range, broader distribution of EDR values, and subsequent heterogeneous distribution of aggregates (Figure 4, row 5). Increased agitation also means greater shear forces, which likely contributes to smaller aggregate diameters.

These observed iPSC aggregate morphologies also confirmed the inverse relationship between agitation rate (and, by extension, average EDR) and aggregate diameter. As seen at 0.5 L (Figure 4, rows 2, 3, 5), increasing agitation and average EDR resulted in small aggregates. Conversely, decreasing agitation and average EDR resulted in much larger and even misshapen aggregates (Figure 4, row 4). Adjusting the agitation rate of VW bioreactors while maintaining average EDR within the optimal range will allow for size control of spherical aggregates during manufacturing scale-up.

The iPSC aggregates expanded in VW bioreactors were tested for expression of pluripotency markers (SSEA-4, TRA-1-60, Klf4, and Nanog), in vitro differentiation potential into tri-lineages, and in vivo functionality (teratomas). The quality test results indicated that pluripotency of the cells was maintained during expansion scale-up [28].

**Figure 5:**
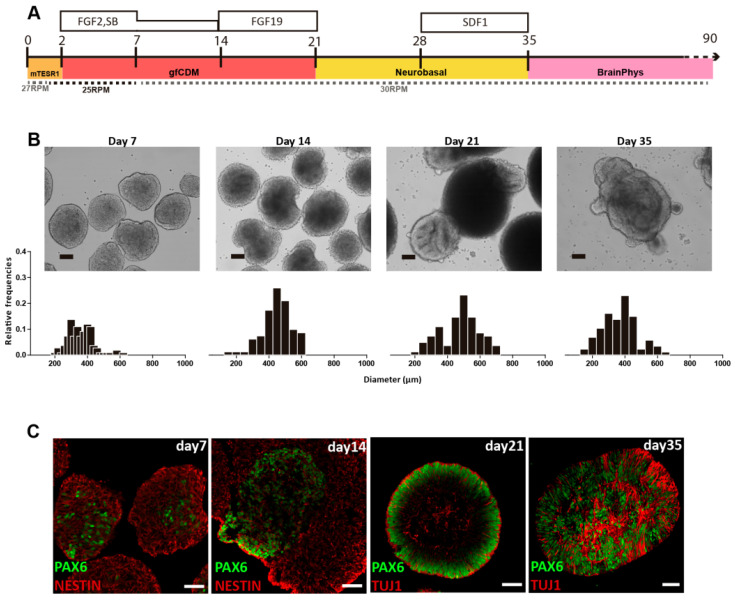
Generation of human iPSC-derived cerebellar organoids using 0.1 L VW bioreactors. (**A**) Process for differentiation of iPSCs to cerebellar organoids. (**B**) Brightfield photomicrographs of spherical iPSC-derived organoids during cerebellar differentiation process. Scale bar: 100 *μ*m. Graphs of organoid diameter distributions indicate homogeneous sizes were maintained. (**C**) Immunofluorescence for NESTIN, PAX6, and TUJ1 during cerebellar differentiation indicate efficient neural induction in iPSC-derived organoids. Scale bar: 50 *μ*m. Adapted from Silva et al. (2020) [29].

The ability of VW bioreactors to consistently manufacture uniform PSC aggregates will be essential for directed differentiation processes at large scales. Spherical iPSC aggregates produced in small-scale VW bioreactors were successfully differentiated into neural cells, which then formed cerebellar organoids in suspension [29]. After 35 days of a cell expansion and differentiation process, iPSC-derived organoids were efficiently differentiated into cerebellar progenitors that further originated mature GABAergic and Glutamatergic neurons (Figure 5). 

By optimizing agitation rate and morphology at each step, the spherical iPSC aggregates promoted even diffusion of growth factors and thus minimized heterogeneous differentiation throughout the 35-day process. While this differentiation was performed at a small scale, CFD modeling has shown that optimal hydrodynamic conditions for differentiation can be maintained during scale-up for commercial production. While the hydrodynamic environment of a bioreactor is a critical parameter, there are other biological requirements of living PSCs that will also affect cell yield and quality at larger scales. 

## 4. Parameters of Dissolved O_2_ and CO_2_

As aerobic organisms, human-derived PSCs consume oxygen and produce carbon dioxide as waste. The requirements of maintaining dissolved oxygen (dO_2_) and dissolved carbon dioxide (dCO_2_) at appropriate levels in the bioreactor’s liquid medium present another scale-up challenge.

The optimal dO_2_ level for a particular PSC culture process needs to be determined during a process optimization study, as different cell types can have different metabolic requirements, even during a multi-step differentiation process. In one study, relatively low levels of dO_2_ (2–9%) were found to be ideal for maintaining the stemness of an hESC line as opposed to recreating ambient air conditions (21% dO_2_) [30,31]. On the other hand, another study reported that a reduced dO_2_ tension (5%) was not beneficial for maintaining different hESCs in an undifferentiated state [32]. Regardless of the required dO_2_ level for a particular cell line, bioreactor design and process conditions must provide an oxygen transfer rate greater than the uptake rate by the cells at peak demand to maintain the required dO_2_ concentration. In addition, the removal of dCO_2_ generated by the cells needs to be at a sufficient rate to prevent negative impacts, such as lowering the pH of liquid medium to a level that may inhibit cell growth. In order to maximize quantity and quality of cells in a bioreactor, dO_2_ replenishment and dCO_2_ stripping should be considered as key control parameters [33]. 

Traditionally in bioreactors, the vessel’s headspace contains a gas-liquid contact surface area through which oxygen can transfer into the liquid medium and dCO_2_ can transfer out. However, gas transfer solely through the headspace gas-liquid surface area is unlikely to be sufficient at larger scales, as the headspace does not typically scale proportionally with liquid medium volume (depending on vessel design). The k_L_a from headspace gassing in a bioreactor decreases at larger working volumes as the gas-liquid interfacial area per liquid volume decreases.

Sparging supplemental oxygen directly into the liquid medium through a port has been used often for traditional *E. coli* or CHO cell culture processes (e.g., for recombinant protein or monoclonal antibody production) and provides both dO_2_ replenishment and dCO_2_ stripping functions. However, sparging gas directly into a liquid medium containing therapeutic cells such as PSC aggregates is unattractive. Of particular concern are the small bubbles generated by the sparging port that rise to the liquid surface layer and create foam or bubbles. Cells can become trapped on the surface of these foam or bubbles and thus be removed from access to needed nutrients and agitation, ultimately resulting in a reduction of total cell yield during a cell culture process. Anti-foaming chemical agents do exist that alleviate the formation of foam, but as hydrophobic agents, they can become incorporated into the membranes of cells. This is of minimal concern for traditional processes where proteins such as monoclonal antibodies are the desired product, and the cells are merely production hosts to be discarded. With cell therapies, the cells themselves are the product, and incorporated anti-foaming chemicals can have unknown effects on cells, posing a potential risk for human patients. Therefore, the addition of anti-foaming agents or similar chemicals is strongly undesirable for cell therapy culture processes. Additionally, bubbles may burst once they reach the surface layer of the liquid medium and cause hydrodynamic shear stress to cells, negatively affecting the viability of cells grown as aggregates or on adherent scaffolds such as microcarriers. The bursting action may also throw the cell aggregates onto parts of the vessel above the liquid level, where they may attach and remain, thus reducing the overall cell yield. Reducing the average size of bubbles introduced into the medium can help reduce the number of bubbles but may exacerbate the problem with the formation of a more stable foam layer. 

Replacing a portion or most of the medium that has been depleted of dO_2_ (and other nutrients) has been another method to provide oxygen to cells. As an example, step-wise removal of some volume of the spent medium after allowing the cells to settle can be followed by the addition of fresh medium. However, this process typically requires a lengthy pause in mixing, which can be detrimental to the growth and quality of PSC aggregates. At larger volumes, the amount of time necessary for cells to settle inevitably increases, exacerbating this problem. 

A potential solution (patent pending) is to retain cells in the bioreactor while continuously removing the dO_2_-depleted medium to an external device or even a second bioreactor. Oxygen enrichment of cell-free medium (up to 476% air saturation) and CO_2_ stripping can be done rapidly by using the external gas exchange device or sparging in the second bioreactor while avoiding gas foaming without worrying about harming cells in the first bioreactor. The dO_2_-rich and dCO_2_-reduced medium may be quickly introduced back into the original bioreactor and circulated at a rate to maintain the bioreactor culture dO_2_ within an acceptable range for the process without a direct sparging in the bioreactor.

Membrane gas exchangers typically utilize diffusion principles, operating much like a shell-and-tube heat exchanger. Oxygen-depleted medium from the bioreactor circulates through one side of the exchanger, with oxygen being pumped into the other side. A highly permeable, biocompatible membrane such as silicon can allow pure oxygen in the gas phase to diffuse into the medium as it circulates through the device. Setting the pump speed used to draw medium from the bioreactor and the oxygen flow rate into the device will determine the level of oxygenation of the medium (up to 476% air saturation) to be introduced back into the reactor. If the specific oxygen consumption rate of the cells cultured in the bioreactor is known, operators can calculate the cell-free medium at a required circulation rate to maintain a sufficient oxygen concentration in the medium in the bioreactor for cell survival and growth.

## 5. Processing Time Related to Medium Exchange

As part of the upstream process, the liquid medium that PSC aggregates are suspended in will need to be exchanged, which involves removing the spent medium and adding fresh medium. Multiple exchanges may be necessary to replenish nutrients, supply specific growth factors and cytokines, and eliminate metabolic wastes and other unwanted media components based on cell culture process requirements.

There are various techniques for performing medium exchange in bioreactors. One common method is to pause agitation and allow all the cells or aggregates to settle by gravity to the bottom of the bioreactor. Once a bed of settled cell aggregates is formed, the supernatant of spent medium is removed, fresh medium is added, and agitation is restarted to resuspend the cells or aggregates. This method has two potential issues, both of which become exacerbated as bioreactor working volume increases. First, the temporary cessation of mixing can decrease cell quality and functionality through unwanted agglomeration, nutrient starvation, and deviation of key process parameters such as temperature, pH, and dO_2_ levels. Second, it is difficult to completely remove all the spent medium, as withdrawing supernatant too close to the bed of settled cell aggregates can result in cell loss, while using a filtered retention device can lead to clumping, clogging, and cell damage. Certain processes, such as multi-step differentiation of PSC aggregates, can have reduced efficiency and yields if the previously used growth factors and cytokines remaining in the spent medium are not completely removed between each differentiation step. Complete settling of cell aggregates after pausing agitation may occur quickly enough to minimize damage at smaller scales. However, complete settling at a commercial scale may take a prolonged time and would severely impact cell yield and quality.

**Figure 6:**
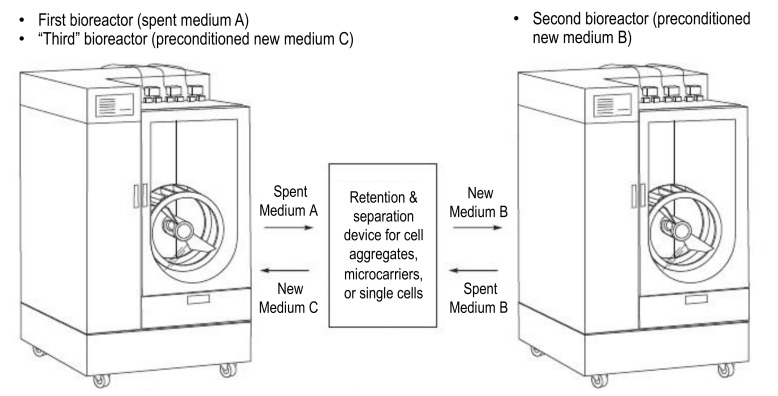
A methodology for using an external retention and separation device in conjunction with multiple bioreactors to facilitate rapid, complete, and scalable medium exchange.

A process that can achieve rapid and complete medium exchange at large volumetric scales would greatly improve the yield and efficiency of processes for PSC expansion and differentiation and thus be an invaluable tool for commercial manufacturing. An example methodology (patent pending) would be to rapidly remove spent medium, containing all or a portion of PSC aggregates, to an external retention and separation device that will concentrate and thoroughly wash cells with fresh medium before transferring them to a different bioreactor that has been prepared with the identical medium used for washing (Figure 6).

After an expansion or differentiation process step is completed in a first bioreactor, spent medium A containing all or a portion of PSCs is quickly transferred to a temperature-controlled separation and retention device where the cells are collected and concentrated while the majority of spent medium is removed. The cells are then washed with the new medium B required for the next process step and then transferred immediately to a second bioreactor, which has already been prefilled with the identical medium B and preconditioned for necessary parameters such as the temperature, pH, and dO_2_. Prefilling and preconditioning of the second bioreactor can begin while the previous step is ongoing in the first bioreactor. After all PSC aggregates have left the first bioreactor, it becomes available to be prefilled and preconditioned with new medium C as the “third” bioreactor in sequence. The now “third” bioreactor should be ready to receive the PSCs after they move from the second bioreactor in spent medium B, into the separation and retention device, and back out of the device. Alternatively, if the first bioreactor cannot be prepared in time, a completely new, third bioreactor can be prefilled and preconditioned instead; any number of bioreactors can be used as dictated by the time required for prefilling and preconditioning a bioreactor. By using the separation and retention device as a bridge to cycle between bioreactors, multiple complete medium exchange steps can be accomplished efficiently and quickly, even at large scales.

## 6. Harvesting Process for Cells

For the expansion of PSC aggregates in bioreactors, a robust cell harvesting procedure (e.g., dissociating PSC aggregates into single cells or small clumps then washing and concentrating) is essential for steps such as serial passaging and final product collection. Compared to harvesting monolayer PSCs grown in 2D planar vessels, recovery of single cells from multilayer PSC aggregates is much more challenging. While recent bioprocess publications have studied in-vessel harvesting of other therapeutic cell types such as mesenchymal stem/stromal cells grown on microcarriers [34,35,36], few studies have examined similar harvesting of PSC aggregates. 

At smaller scales, separating cell aggregates and culture medium may be achieved through gravitational settling of the aggregates and removing the medium from the supernatant. As explained previously in regard to a medium exchange, complete settling at larger scales would take so long as to decrease cell yield and quality. Since a rapid and complete medium exchange is not necessary during harvesting, more scalable options are available such as staggered removal of medium from different heights of the bioreactor, splitting all cell-containing medium into multiple vessels to shorten the settling time in each, or even using the retention device previously described for medium exchange.

One or two wash steps may be necessary during the cell harvest process, with the main purpose of removing excess proteins that could inhibit enzyme-based cell dissociation. During each wash step, an appropriate buffer such as basal media or Mg^−^ and Ca^−^ DPBS is added to resuspend and rinse cell aggregates. The wash buffer may also need to be removed via medium exchange prior to cell dissociation if a high concentration of dissociation enzyme was used, adding another process step and potential for cell loss.

Once the culture medium has been removed, and cells have been washed, a dissociation protocol is required to break up large cell aggregates into viable single cells for serial passaging or final product collection. In most cases, a combination of dissociation enzymes and agitation is used. Simply increasing both the amount of added enzymes and agitation rate may speed up dissociation but also cause other issues. The processing time to remove excess enzymes may be increased, and agitation should not be so high that the resulting shear forces potentially damage cells on the surface of PSC aggregates. Ideally, a harvesting protocol encompassing separation, washing, and dissociation should be optimized at a small scale before scaling up to large-scale bioreactors. 

The first published protocol for in-vessel aggregate dissociation of iPSC aggregates was performed in a 0.1 L VW bioreactor [37]. This study tested various enzyme types and dissociation times. An optimized protocol was developed that utilizes Accutase^®^ for a 20-min period at 80 rpm and achieves over 95% harvest recovery with greater than 90% viability. Importantly, this study demonstrated that the harvested cells could be inoculated as single cells in both 0.1 L and 0.5 L VW bioreactors and reform high-quality iPSC aggregates. The cells maintained consistent growth kinetics during bioreactor passages and normal karyotype and pluripotency as demonstrated through tri-lineage differentiation assays following serial passaging. Moreover, gene expression analysis by RT-qPCR demonstrated comparable or higher pluripotency-associated genes (Oct-4, Nanog, Rex1, Sox2, and Klf4) in the harvested iPSCs following serial passaging in VW bioreactors compared to those passaged in static culture controls [37]. With the predictable hydrodynamic conditions of VW bioreactors, this protocol will be scaled up and optimized for large-volume harvesting. 

## 7. Conclusions

The goal of reliably providing PSC-derived allogeneic cell therapies to vast numbers of patients requires a series of optimized unit operations at various scales to meet target manufacturing lot sizes. Numerous manufacturing processes such as cell aggregate expansion and differentiation, gas and medium exchanges, and cell harvesting need to be developed and optimized for large-scale use. The proper combination of single-use bioreactor technology and methodologies can avoid potential upstream process bottlenecks and enable robust commercial-scale manufacturing of therapeutic cells. 

## Data Availability

The data presented in this study are available on request from the corresponding author.
